# Childhood and adolescent phenol and phthalate exposure and the age of menarche in Latina girls

**DOI:** 10.1186/s12940-018-0376-z

**Published:** 2018-04-03

**Authors:** Alexandra M. Binder, Camila Corvalan, Antonia M. Calafat, Xiaoyun Ye, Verónica Mericq, Ana Pereira, Karin B. Michels

**Affiliations:** 1 Department of Epidemiology, Fielding School of Public Health, University of California, Los Angeles, CA USA; 2 Institute of Nutrition and Food Technology, University of Chile, Santiago, Chile; 3 Division of Laboratory Sciences, National Center for Environmental Health, Centers for Disease Control and Prevention, Atlanta, GA USA; 4 Institute of Maternal and Child Research, University of Chile, Santiago, Chile

**Keywords:** Menarche, Puberty, Phenols, Phthalates, Endocrine disrupting chemicals

## Abstract

**Background:**

The age of menarche has been associated with metabolic and cardiovascular disease, as well as cancer risk. The decline in menarcheal age over the past century may be partially attributable to increased exposure to endocrine disrupting chemicals (EDCs).

**Methods:**

We assessed the influence of 26 phenol and phthalate biomarkers on the timing of menarche in a longitudinal cohort of Chilean girls. These EDCs were quantified in urine collected prior to the onset of breast development (Tanner 1; B1), and during adolescence (Tanner 4; B4). Multivariable accelerated failure time (AFT) models were used to analyze associations between biomarker concentrations and the age of menarche adjusting for body mass index (BMI) Z-score and maternal education, accounting for within-subject correlation.

**Results:**

Several biomarkers were significantly associated with the age at menarche; however, these associations were dependent on the timing of biomarker assessment. A log(ng/ml) increase in B1 concentrations of di(2-ethylhexyl) phthalate biomarkers was associated with later menarche (hazard ratio (HR): 0.77; 95% CI: 0.60, 0.98), whereas higher B1 concentrations of 2,5-dichlorophenol and benzophenone-3 were associated with earlier menarche (HR: 1.13; 95% CI: 1.01, 1.27; HR: 1.17; 95% CI: 1.06, 1.29, respectively). Elevated B4 concentrations of monomethyl phthalate were similarly associated with earlier menarche (HR: 1.30; 95% CI: 1.10, 1.53). The impact of monoethyl phthalate and triclosan concentrations on pubertal timing were significantly modified by BMI Z-score. Higher monoethyl phthalate and triclosan concentrations were associated with earlier menarche among overweight or obese girls, but not among those that were normal weight.

**Conclusions:**

This study identifies modulation of sexual maturation by specific EDC biomarkers in Latina girls.

**Electronic supplementary material:**

The online version of this article (10.1186/s12940-018-0376-z) contains supplementary material, which is available to authorized users.

## Background

Determinants of pubertal timing are of public health concern because of the association between the age of onset and adult disease susceptibility. Among females, early menarche has been associated with an increased risk of type 2 diabetes, cardiovascular disease, and reproductive cancers, including breast [1–3]. The average age of menarche has decreased from 16-17 years at the end of the nineteenth century, to less than 13 years across Europe and the United States, likely due to improvements in nutrition and general health [4, 5]. While the age of menarche was thought to have stabilized in the past 50 years, more recent studies have suggested that the age of menarche has continued to decline in South America, the United States, and many countries in Europe [6–9]. The secular trend towards earlier menarche has been observed across race/ethnicity groups, despite differences in timing [7, 10–12]. Within the United States, the average age of menarche is earlier among Hispanic and black females relative to white females, adjusting for BMI, as well as social and economic indicators [7, 13]. Across Europe and South America, the fastest decline has been among impoverished girls, in spite of earlier menarche being previously more common among affluent families [6, 8, 9, 14, 15].

The two primary hypotheses for this shift towards earlier menarche are the growing childhood obesity epidemic and increasing exposure to endocrine disruptor chemicals (EDCs) [16–18]. EDCs can influence the endocrine system through a multitude of mechanisms, including competitive binding to hormone receptors to modulation of hormone synthesis and metabolism [19]. There is building evidence that EDCs can affect proper function across a number of different tissues, including breast tissue and female reproductive organs [19], as well as influencing the neuroendocrine control of reproduction. Results of both in vivo studies and a few longitudinal human cohorts suggest some of these compounds may be obesogenic, with early life exposure associated with childhood body size [20–23]. Phenols and phthalates are two classes of EDCs of particular concern due to their use in an extensive array of products, including plastics, building materials, personal care products, insecticides, and herbicides [24]. In this study, we analyzed the association between pre-pubescent and pubescent biomarkers of exposure to select phenols and phthalates and the timing of menarche within a longitudinal cohort of Chilean girls born in 2002–2003. Two cross-sectional studies and four longitudinal studies have analyzed the influence of childhood EDC exposure on the age of menarche [25–30]. The results of these studies have been largely discordant, potentially reflecting regional and socioeconomic influences on exposure profile, differences in the age of biomarker measurement, and possible effect modification by race/ethnicity. For this study, we quantified a broad panel of 26 phenol and phthalate urinary biomarker concentrations prior to the onset of breast development (Tanner 1; B1) and at Tanner 4 (B4). This study provides unique insight into the influence of these compounds on pubertal timing in Latina girls.

## Methods

### Study population

Our study population was a random subset of 200 girls part of the longitudinal Growth and Obesity Cohort Study (GOCS) with urine samples collected at B1 (ages 6.7 to 9.6 years; median age: 7.9 years) and B4 (ages 9.4 to 13.1 years; median age: 11.2 years). The GOCS children were born in 2002–2003, and are representative of the low to middle-income families in Santiago, Chile. A description of the cohort has been provided elsewhere [31]. Starting in 2009, breast development was assessed by two trained dietitians (kappa with pediatric endocrinologist = 0.85) by visual inspection using Tanner’s rating scale approximately every 6 months [32]. Palpation was additionally used to distinguish breast Tanner 1 from Tanner 2. Sex and age-adjusted BMI Z-score at each visit were calculated based on the Centers for Disease Control and Prevention (CDC) growth charts. Girls were categorized as having excess weight (overweight or obese) if their BMI Z-score was equal or above the 85th percentile. Informed consent was obtained from all parents or guardians of children before the start of data collection.

### Biomarker measurements

Fasting spot urine samples were collected between 10 AM and 12 PM in polypropylene sterile cups, and were immediately vortexed and aliquoted. Concentrations of 26 phenol and phthalate biomarkers were quantified in urines collected at breast Tanner 1 (B1) and Tanner 4 (B4) from 200 girls (400 samples). The analytical measurements were performed at the CDC National Center for Environmental Health Laboratory using previously described analytical methods [33, 34]. Concentrations below the limit of detection (LOD) were given an imputed value equal to LOD/sqrt(2) [35]. Biomarker concentrations (ng/ml) were corrected for specific gravity. Dilution adjustment was performed using the formula P_c_ = P[(1.015–1)/(SG-1)], where P_c_ is the specific gravity-corrected biomarker concentration, P is the observed biomarker concentration, SG is the specific gravity of the urine sample, and 1.015 is the median SG of the study population [36, 37]. To calculate the summation of di(2-ethylhexyl) phthalate (DEHP) metabolites (⅀DEHP), concentrations of MEHP, MEOHP, MEHHP, and MECPP were converted to nmol/L before being added together. The analysis of blinded specimens by the CDC laboratory was determined not to constitute engagement in human subjects’ research.

### Age of menarche

Prior to the onset of B4, girls were asked to report the date of their first menstrual bleeding at each 6-month visit. After achieving B4, girls were contacted by study dieticians every 3 months to survey whether the girl had reached menarche. During this phone interview, a questionnaire was used to differentiate menarche from other potential causes of vaginal bleeding, such as vaginal infection, urinary infection or trauma. Longitudinal follow-up of participants enabled the confirmation of menarche onset.

### Statistical methods

Multivariable accelerated failure time (AFT) models were used to assess the influence of individual phenol and phthalate biomarker concentrations on time to menarche, assuming a Weibull distribution. To account for possible confounding by body size, models were adjusted for BMI Z-score at EDC measurement [16–18, 38, 39]. Models were additionally adjusted for maternal education as an indicator of socioeconomic status, which is related to both exposure profile and age of menarche [6, 8, 9, 14, 15, 40]. Sensitivity analyses were conducted further adjusting for the mother’s age of menarche (recall; ≤11, 12, 13,≥14 years), which may capture confounding by transgenerational exposures correlated with socioeconomic status. These analyses were restricted to the subset of the families that reported mother’s age of menarche (*N* = 181), assuming recall data was missing completely at random. A cluster statement was used to account for within-subject correlation between B1 and B4 biomarker measurements. Accordingly, inference was based on robust standard errors estimated using the Huber sandwich estimator. For incident cases, survival time was the age at menarche, estimated based on time between the self-reported date of first menses and date of birth. Survival time for right censored individuals was the age at last clinic visit, based on the time between date of last visit and date of birth. Time-varying associations between biomarker concentration (log(ng/ml)) and the age of menarche were investigated by adding Tanner stage as well as an interaction between biomarker concentrations and Tanner stage to our models. Association estimates from the AFT models were reported as the relative change in hazard of menarche (hazard ratio; HR) associated with a log(ng/ml) increase in biomarker concentration, which generally corresponded to a change from the lowest concentration quartile to the highest quartile. If the interaction between Tanner stage and concentration on timing of menarche was significant (Wald test; *p* < 0.05), the reported associations were stratified by Tanner stage. Similarly, a Wald test was used to assess effect modification by BMI Z-score. If the interaction term significantly improved model fit (p < 0.05), the influence of the biomarker on the timing of menarche was reported separately for overweight/obese girls and normal weight girls. To identify potential non-monotonic dose-response relationships, we additionally modeled tertiles of biomarker concentration, with tertile cut-points stratified by Tanner stage. Significant trends across categories were evaluated by modeling the log(median) concentration within tertiles as a continuous variable. To visualize the change in the timing of menarche, we computed median age at menarche for tertiles of urinary concentrations using the baseline survivor function of multivariable adjusted AFT models. All statistical analysis was performed in R Version 3.3.1 and figures were generated using ggplot2 [41].

## Results

The influence of EDC exposure across childhood and adolescence on the age of menarche was assessed among 200 Chilean girls born in 2002–2003. The median age at urine collection prior to the onset of breast development (B1) was 7.9 years and 11.2 years at Tanner 4 (Table 1). Proportion of samples with measurements above the limit of detection, as well as the geometric mean (95% CI) biomarker concentrations at B1 and B4 are reported in Additional file 1: Tables S1a and S1b. Analysis was restricted to the subset of 21 biomarkers with detectable concentrations at Tanner 1 and Tanner 4 in at least 75% of the girls. These 21 biomarkers included: 2,4-dichlorophenol, 2,5-dichlorophenol, benzophenone-3, BPA, MBP, MBzP, MCNP, MCOP, MCPP, MECPP, MEHHP, MEHP, MEOHP, MEP, methyl paraben, MHBP, MHiBP, MiBP, MMP, propyl paraben, triclosan; excluded were: MNP, B-PB, BPF, BPS, and E-PB (see Abbreviation List; Additional file 1: Table S1a and S1b). For reference, geometric mean (95% CI) phenol and phthalate creatine-adjusted urinary biomarker concentrations among U.S. females of a similar age range from the 2011–2012 National Health and Nutrition Examination Survey are reported in Additional file 1: Table S2. In both the U.S. and Chilean populations, most biomarker concentrations decreased with age (Additional file 1: Table S1a and S1b; Additional file 1: Table S2). The intra-individual correlation between phenol and phthalate biomarkers at B1 and B4 was moderate to low (Additional file 1: Table S3). The mean age of menarche was 12.0 years; the median age was similarly 12.0 years (95% CI: 11.9–12.2 years). At the time of analysis, 18 girls had not reached menarche or had dropped out of the cohort. Of those that achieved menarche during the course of the study, 10.5% (*n* = 21) reached menarche prior to the first detection of B4. The occurrence of menarche prior to B4 did not significantly modify the linear association between any biomarkers and the age of menarche in adjusted models. Therefore, B4 biomarker measurements for these 21 girls were included in all subsequent models. Biomarker concentrations at B1 were not significantly correlated with BMI Z-score. Similarly, most B4 biomarker concentrations were not correlated with B4 BMI Z-score, with the exception of monoethyl phthalate (MEP) and 2,5-dichlorophenol, which had moderate positive associations with BMI Z-score (ρ = 0.21 and ρ = 0.22, respectively). Additional study population characteristics are outlined in Table 1.

**Table 1:** Study population characteristics

Characteristic	Distribution
Age [years; median (range)]	
At B1 EDC Measurement	7.9 (6.7–9.6)
At B4 EDC Measurement	11.2 (9.4–13.1)
Attained Menarche during Study Category [count (%)]	
Yes	182 (91.0)
No	18 (9.0)
Age at Menarche [years; median (range)]	12.0 (9.4–13.8)
BMI Z-score [median (range)]	
At B1 EDC Measurement	0.5 (−2.2–2.4)
At B4 EDC Measurement	0.5 (−2.7–2.2)
BMI Category [count (%)]	
At B1 EDC Measurement	
Under-weight or Normal weight (<85th Percentile)	147 (73.5)
Overweight or Obese (≥85th percentile)	53 (26.5)
At B1 EDC Measurement	
Under-weight or Normal weight (<85th Percentile)	137 (68.5)
Overweight or Obese (≥85th percentile)	63 (31.5)
Height Z-score [median (range)]	
At B1 EDC Measurement	−0.1 (−2.6–2.1)
At B4 EDC Measurement	0.1 (−2.1–2.3)
Maternal Education [count (%)]	
Post-Secondary Education	46 (23.0)
No Post-Secondary Education	154 (77.0)

**Fig. 1:**
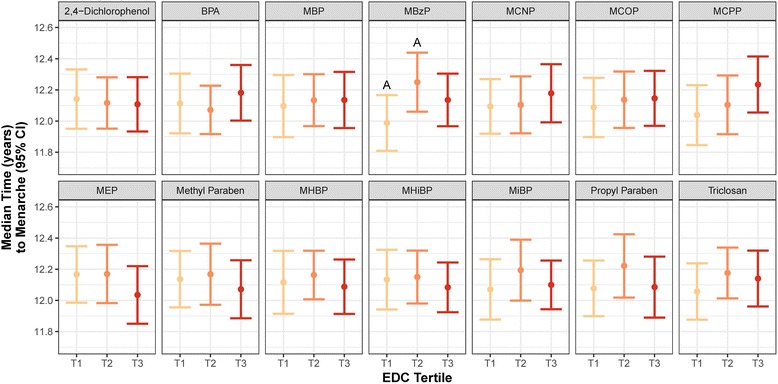
Median menarcheal age (95% CI) between tertiles of EDC biomarker concentrations across Tanner stages. Significant difference (*p* < 0.05) between: tertiles 1 and 2 indicated by A, tertiles 2 and 3 indicated by B, and tertiles 1 and 3 indicated by C

We did not detect a significant linear impact of average concentration across Tanner stages on the age of menarche for any biomarker (Table 2). However, the association between urinary biomarker concentrations and the timing of menarche was significantly modified by the Tanner stage at biomarker measurement for 2,5-dichlorophenol, benzophenone-3, monomethyl phthalate (MMP), and all of the metabolites of DEHP after adjusting for BMI Z-score and maternal education (Table 2). Further adjustment for mother’s age of menarche (≤11, 12, 13, ≥14 years) did not change the shape or strength of the associations between EDC biomarker levels and daughter’s age of menarche (Additional file 1: Tables S4 and S5). For ease of interpretation, and to identify potential non-linear influences, we evaluated the association between tertiles of each biomarker and the timing of menarche in adjusted models (Table 3; Fig. 1; T1=lowest tertile, T2=middle tertile, T3=highest tertile). Among the subset of biomarkers for which we did not detect significant effect modification by Tanner stage at measurement, we found a non-monotonic change in the timing of menarche between tertiles of monobenzyl phthalate (MBzP) across puberty (Table 3; Fig. 1). While there was a later age of menarche among the second tertile of MBzP relative to the first (HR: 0.70; 95% CI: 0.54, 0.93), the highest tertile was not significantly different from the lowest (Table 3; Fig. 1).

**Table 2:** Menarche hazard ratio (95% CI) associated with log(ng/ml) increase in each EDC biomarker across puberty

Biomarker	Unadjusted Model	Adjusted Model^a^	Interaction with Tanner stage^b^	Interaction with BMI^c^
2,4-Dichlorophenol	0.98 (0.87, 1.10)	1.00 (0.89, 1.12)	0.246	0.062
2,5-Dichlorophenol	1.05 (0.97, 1.14)	1.04 (0.95, 1.14)	0.017*	0.975
Benzophenone-3	1.03 (0.97, 1.09)	1.05 (0.99, 1.11)	0.009**	0.235
BPA	0.90 (0.78, 1.05)	0.92 (0.79, 1.06)	0.362	0.790
MBP	0.95 (0.84, 1.07)	0.96 (0.85, 1.08)	0.565	0.396
MBzP	0.98 (0.87, 1.09)	0.94 (0.84, 1.05)	0.729	0.891
MCNP	0.95 (0.82, 1.10)	0.91 (0.79, 1.05)	0.392	0.797
MCOP	0.95 (0.81, 1.10)	0.92 (0.78, 1.09)	0.638	0.790
MCPP	0.91 (0.80, 1.04)	0.91 (0.79, 1.05)	0.157	0.252
ΣDEHP^d^	1.04 (0.88, 1.22)	1.02 (0.87, 1.19)	0.002**	0.783
MECPP	1.05 (0.89, 1.24)	1.02 (0.87, 1.20)	0.004**	0.855
MEHHP	1.03 (0.89, 1.18)	1.01 (0.88, 1.16)	0.002**	0.643
MEHP	1.01 (0.85, 1.20)	1.01 (0.85, 1.20)	0.000***	0.909
MEOHP	1.02 (0.88, 1.19)	1.01 (0.87, 1.16)	0.004**	0.852
MEP	1.10 (0.99, 1.23)	1.09 (0.98, 1.21)	0.177	0.033*
Methyl Paraben	1.00 (0.94, 1.07)	1.00 (0.94, 1.07)	0.861	0.576
MHBP	1.02 (0.89, 1.16)	1.02 (0.89, 1.17)	0.486	0.488
MHiBP	1.04 (0.88, 1.23)	1.04 (0.88, 1.24)	0.426	0.920
MiBP	1.02 (0.87, 1.19)	1.01 (0.86, 1.19)	0.955	0.671
MMP	1.05 (0.93, 1.19)	1.08 (0.97, 1.22)	0.017*	0.262
Propyl Paraben	0.99 (0.92, 1.05)	0.99 (0.93, 1.05)	0.495	0.160
Triclosan	0.98 (0.90, 1.07)	1.00 (0.92, 1.08)	0.956	0.033*

^a^Accelerated failure time model adjusting for BMI Z-score, and maternal education

^b^*p*-value for interaction between continuous biomarker concentration and Tanner stage at biomarker measurement in adjusted models

^c^*p*-value for interaction between continuous biomarker concentration and BMI Z-score in adjusted models

^d^units in log(nmol/l) reflecting the log transformed summation of DEHP metabolite concentrations

*p < 0.05, **p < 0.01, ***p < 0.001

**Table 3:** Menarche hazard ratio (95% CI) between tertiles of each EDC biomarker across puberty^a^

Biomarker	Middle vs Lowest Tertile	Highest vs Middle Tertile	Highest vs Lowest Tertile	Trend^b^ (*p*-value)
2,4-Dichlorophenol	1.03 (0.79, 1.35)	1.01 (0.79, 1.30)	1.04 (0.80, 1.37)	0.701
BPA	1.06 (0.81, 1.38)	0.86 (0.67, 1.11)	0.91 (0.70, 1.20)	0.695
MBP	0.95 (0.74, 1.23)	1.00 (0.76, 1.31)	0.95 (0.69, 1.31)	0.749
MBzP	0.70* (0.54, 0.93)	1.16 (0.88, 1.55)	0.82 (0.62, 1.08)	0.155
MCNP	0.99 (0.77, 1.27)	0.91 (0.67, 1.23)	0.89 (0.67, 1.19)	0.433
MCOP	0.94 (0.72, 1.22)	0.99 (0.75, 1.30)	0.93 (0.68, 1.27)	0.661
MCPP	0.92 (0.67, 1.26)	0.84 (0.63, 1.13)	0.77 (0.57, 1.04)	0.116
MEP	1.00 (0.74, 1.34)	1.20 (0.88, 1.62)	1.19 (0.90, 1.57)	0.212
Methyl Paraben	0.96 (0.71, 1.29)	1.14 (0.84, 1.54)	1.09 (0.81, 1.47)	0.575
MHBP	0.94 (0.72, 1.23)	1.10 (0.88, 1.39)	1.04 (0.76, 1.41)	0.808
MHiBP	0.98 (0.76, 1.26)	1.09 (0.86, 1.39)	1.07 (0.82, 1.39)	0.654
MiBP	0.85 (0.63, 1.15)	1.13 (0.85, 1.50)	0.96 (0.74, 1.26)	0.763
Propyl Paraben	0.83 (0.60, 1.13)	1.20 (0.85, 1.68)	0.99 (0.73, 1.33)	0.918
Triclosan	0.85 (0.66, 1.10)	1.05 (0.84, 1.30)	0.90 (0.67, 1.20)	0.536

^a^Accelerated failure time model adjusting for BMI Z-score and maternal education; restricting to the subset of associations for which there was no significant interaction between Tanner stage at biomarker measurement and biomarker concentration on timing of menarche (*p* > 0.05)

^b^Trend evaluated by modeling the log(median) concentration within tertiles as a continuous variable

*p < 0.05, **p < 0.01, ***p < 0.001

**Fig. 2:**
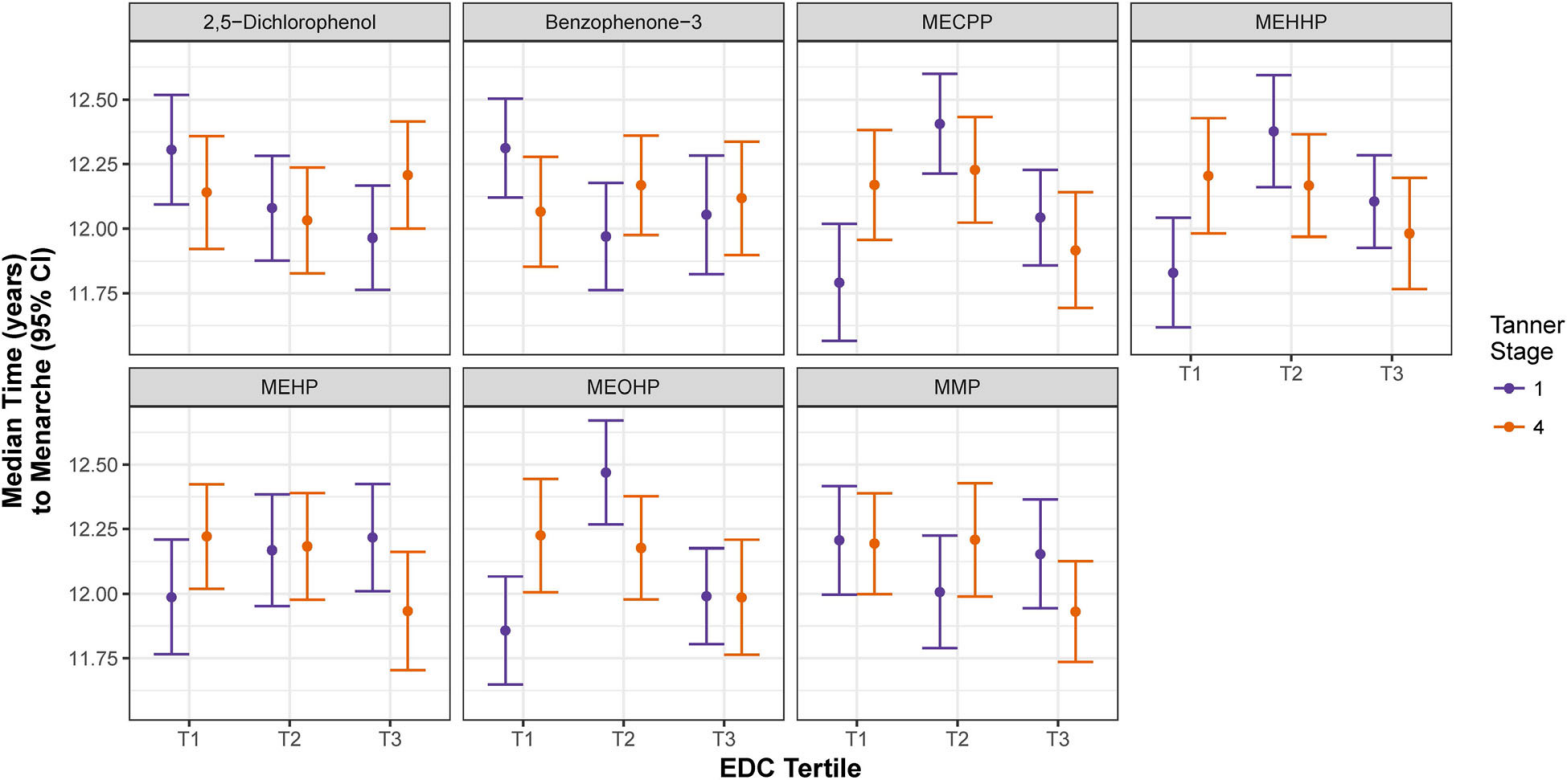
Median menarcheal age (95% CI) between tertiles of EDC biomarker concentrations stratified by Tanner stage

Increased B1 concentrations of 2,5-dichlorophenol and benzophenone-3 were associated with decreased time to menarche (Table 4). In contrast, higher B1 concentrations of the DEHP biomarkers mono(2-ethyl-5-hydroxyhexyl) phthalate (MEHHP), mono(2-ethyl-5-oxohexyl) phthalate (MEOHP), and mono(2-ethylhexyl) phthalate (MEHP) were associated with later menarche (Table 4). Girls in the highest tertile of B1 2,5-dichlorophenol concentration had an earlier age of menarche compared to those in the lowest tertile (Table 5; HR: 1.58; 95% CI: 1.09, 2.29), corresponding to 4.09 months earlier median age of menarche (Fig. 2). The influence of benzophenone-3 and the DEHP metabolites on the relative time to menarche was less linear (Table 5; Fig. 2). The median age of menarche was 4.10 months earlier among girls in the second tertile of B1 benzophenone-3 concentration compared to the lowest tertile (HR: 1.58; 95% CI: 1.12, 2.22), but the change in timing was not significant between the lowest and highest tertiles (Table 5). For all of the secondary oxidative DEHP biomarkers quantified (MEHHP, MECPP, and MEOHP), the median age of menarche was 6.57 to 7.37 months later among girls in the middle tertile relative to those in the lowest tertile of B1 concentration (Table 5). For MEHHP, girls in the highest tertile at B1 also had a significantly later age of menarche, but the median delay was not as great as that among girls in the second tertile (Table 5; Fig. 2).

**Table 4:** Menarche hazard ratio (95% CI) associated with log(ng/ml) increase in biomarker stratified by Tanner stage^a^

	Tanner Stage	
Biomarker	B1	B4
2,5-Dichlorophenol	1.13* (1.01, 1.27)	0.96 (0.85, 1.08)
Benzophenone-3	1.17** (1.06, 1.29)	0.99 (0.91, 1.06)
ΣDEHP^b^	0.77* (0.60, 0.98)	1.24 (0.97, 1.57)
MECPP	0.79 (0.61, 1.01)	1.24 (0.97, 1.58)
MEHHP	0.77* (0.62, 0.96)	1.22 (0.98, 1.51)
MEHP	0.80* (0.65, 0.98)	1.20 (0.98, 1.47)
MEOHP	0.78* (0.63, 0.97)	1.20 (0.96, 1.50)
MMP	0.96 (0.81, 1.14)	1.30** (1.10, 1.53)

^a^Accelerated failure time model adjusting for BMI Z-score and maternal education; including an interaction between Tanner stage at biomarker measurement and concentration; restricted to subset of associations for which the interaction with the timing of biomarker measurement was significant (*p* < 0.05)

^b^units in log(nmol/l) reflecting the log transformed summation of DEHP metabolite concentrations

*p < 0.05, **p < 0.01, ***p < 0.001

**Table 5:** Menarche hazard ratio (95% CI) between tertiles of biomarker stratified by Tanner stage at measurement^a^

Biomarker	Tanner Stage	Middle vs Lowest Tertile	Highest vs Middle Tertile	Highest vs Lowest Tertile	Trend^b^ (p-value)
2,5-Dichlorophenol					
	1	1.35 (0.93, 1.98)	1.17 (0.81, 1.68)	1.58* (1.09, 2.29)	0.019*
	4	1.16 (0.79, 1.69)	0.79 (0.55, 1.14)	0.91 (0.62, 1.35)	0.521
Benzophenone-3					
	1	1.58** (1.12, 2.22)	0.89 (0.60, 1.34)	1.41 (0.96, 2.06)	0.142
	4	0.87 (0.61, 1.24)	1.07 (0.74, 1.54)	0.93 (0.63, 1.37)	0.743
ΣDEHP					
	1	0.43*** (0.31, 0.61)	1.56* (1.09, 2.25)	0.67* (0.46, 0.98)	0.140
	4	0.93 (0.64, 1.36)	1.52* (1.01, 2.28)	1.42 (0.93, 2.17)	0.083
MECPP					
	1	0.43*** (0.30, 0.62)	1.63** (1.15, 2.33)	0.71 (0.47, 1.05)	0.232
	4	0.92 (0.64, 1.34)	1.53* (1.02, 2.31)	1.41 (0.93, 2.15)	0.082
MEHHP					
	1	0.47*** (0.33, 0.68)	1.44 (1.00, 2.09)	0.68* (0.47, 0.99)	0.064
	4	1.05 (0.73, 1.52)	1.29 (0.87, 1.90)	1.36 (0.89, 2.06)	0.148
MEHP					
	1	0.78 (0.54, 1.14)	0.94 (0.64, 1.38)	0.73 (0.50, 1.09)	0.122
	4	1.05 (0.74, 1.49)	1.40 (0.93, 2.10)	1.47 (0.98, 2.20)	0.061
MEOHP					
	1	0.43*** (0.30, 0.62)	1.92*** (1.34, 2.75)	0.83 (0.59, 1.17)	0.313
	4	1.07 (0.73, 1.55)	1.30 (0.87, 1.95)	1.39 (0.91, 2.12)	0.120
MMP					
	1	1.31 (0.89, 1.93)	0.82 (0.55, 1.22)	1.07 (0.74, 1.56)	0.672
	4	0.98 (0.68, 1.42)	1.45* (1.01, 2.10)	1.42* (1.02, 1.99)	0.032*

^a^Estimating Tanner stage-specific association between biomarker and age of menarche adjusting BMI Z-score and maternal education, including an interaction between biomarker tertile and Tanner stage; restricted to subset of associations for which the interaction with Tanner stage was significant (p < 0.05)

^b^Trend evaluated by modeling the log(median) concentration within tertiles as a continuous variable

*p < 0.05, **p < 0.01, ***p < 0.001

A log(ng/ml) increase in B4 concentrations of MMP was associated with a faster time to menarche (Table 4; HR: 1.30; 95% CI: 1.10, 1.53). The influence of B4 concentrations of MMP on the age of menarche was relatively linear. Among girls in the highest B4 concentration tertile, the median age of menarche was 3.15 months earlier than girls in the lowest MMP tertile (Table 5; HR: 1.42; 95%: 1.02, 1.99).

**Fig. 3:**
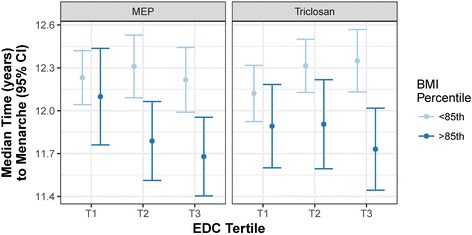
Median menarcheal age (95% CI) between tertiles of EDC biomarker concentrations stratified by BMI percentile

For MEP and triclosan, the influence on the age of menarche was significantly modified by age and sex-adjusted BMI Z-score (Table 2). The average effect of triclosan across various body sizes was negligible, whereas there was a shift towards earlier menarche among girls with higher concentrations of MEP (Table 2). Stratifying by BMI category, a log(ng/ml) increase in triclosan concentration was associated with earlier age of menarche (Table 6; HR: 1.16; 95% CI: 1.01, 1.34) among girls that were either overweight or obese (≥85th percentile BMI Z-score). Similarly, a log(ng/ml) increase in MEP among overweight or obese girls was associated with earlier menarche (HR: 1.24; 95%CI: 1.05, 1.47), with the median age of menarche 5.03 months earlier in the highest compared to the lowest tertile (Fig. 3).There was no significant association with either triclosan or MEP among girls that were normal weight.

**Table 6:** Menarche hazard ratio (95% CI) associated with log(ng/ml) increase of biomarker stratified by BMI percentile^a^

	BMI Percentile	
Biomarker	<85th	≥85th
MEP	1.02 (0.90, 1.17)	1.24* (1.05, 1.47)
Triclosan	0.93 (0.84, 1.02)	1.16* (1.01, 1.34)

^a^Accelerated failure time model adjusting for BMI Z-score and maternal education; restricted to subset of associations for which the interaction with BMI Z-score was significant (p < 0.05)

*p < 0.05, **p < 0.01, ***p < 0.001

## Discussion

In this study, we report changes in the timing of menarche associated with earlier childhood and adolescent biomarker urinary concentrations of several phthalates and phenols. For most of these biomarkers, the strength of the association was dependent on the timing of biomarker measurement, suggesting specific periods of susceptible hormone-driven development. High childhood (Tanner 1) urinary concentrations of DEHP metabolites were associated with later menarche. In contrast, increased childhood levels of 2,5-dichlorophenol and benzophenone-3 were associated with early menarche. Increased adolescent (Tanner 4) concentrations of MMP were similarly associated with earlier menarche. This sensitivity to exposure window may partially explain some discordance between prior studies of childhood EDC exposure and the age of menarche, which evaluated biomarker concentrations at various ages [25–28].

Among this cohort of Latina girls, increased urinary concentrations of DEHP biomarkers during childhood were associated with later menarcheal age. High pre-pubertal concentrations of these biomarkers have been associated with decreased concentrations of adolescent androgens, suggesting DEHP may inhibit adrenal androgen synthesis in humans [29]. This anti-androgenic influence of DEHP is further supported by the later age of pubarche among girls with relatively high concentrations of DEHP metabolites [42, 43]. Another mechanism by which childhood DEHP exposure may influence pubertal timing is through its influence on concentrations of IGF-1 and thyroid hormones [44–47]. DEHP may also act by modifying the production of luteinizing hormone and follicle-stimulating hormone, reducing estrogen formation, or through the activation of peroxisome proliferation-activated receptors [48]. Similar to our findings, one longitudinal study of German adolescents recently reported that higher urinary concentrations of DEHP metabolites from ages 8–10 years were associated with decreased odds of having reached menarche at subsequent visits [30]. Other studies have not reported the same association between urinary DEHP biomarker concentrations and age of menarche. A cross-sectional study of U.S. girls 12–16 years reported the summation of phthalate urinary biomarker concentrations was not associated with the onset of menarche [27]. A smaller cross-sectional study of Hispanic girls in Mexico City also did not report a significant influence of these exposures [28]. In contrast, greater concentrations of DEHP biomarkers between 7 and 15 years were associated with increased odds of earlier menarche within a cohort of Chinese girls [26]. Our study results suggest that the influence of the biomarkers was modified by pubertal stage at the time of exposure assessment, and suggested the correlation in DEHP biomarker concentrations across puberty was weak. Therefore, these disparate findings may partially reflect differences in exposure window.

In contrast to the influence of the DEHP biomarkers, increased concentrations of 2,5-dichlorophenol, benzophenone-3, and MMP were associated with earlier menarche. 2,5-dichlorophenol is the major biomarker of *para*-dichlorobenzene, a chemical used in moth repellants, room deodorants, and fumigants. Two prior studies of U.S. girls reported a similar association between 2,5-dichlorophenol and age of menarche, which is consistent with the estrogenic activity 2,5-dichlorophenol has demonstrated in vitro and in vivo [25, 27, 49]. The sunscreen agent benzophenone-3 has also been shown to have estrogenic characteristics, including exerting uterotrophic effects in rats, simulating proliferation of breast cancer cell lines, as well as binding and activating estrogen receptors [50, 51]. While these compounds may have a similar mechanism of action, prior studies did not report a significant association between benzophenone-3 and menarcheal age [25, 27]. In our study, only pre-pubertal concentrations of 2,5-dichlorophenol and benzophenone-3 were associated with earlier menarche. Conversely, MMP concentrations close to the onset of menarche were associated with earlier timing. Two studies of Chinese girls reported conflicting associations between MMP concentrations and pubertal timing [52, 53]. The parent compound of MMP, dimethyl phthalate (DMP), can be found in insect repellants and plastics. While no estrogenic effect of DMP has been demonstrated in vivo, concentrations of MMP among children 5–7 years have been inversely associated with concentrations of IGF-1, with no significant impact on thyroid function [54]. The potential influence on the regulation of growth and development suggests the need for further investigation into the impact of MMP, or its precursor DMP, on adolescent development.

BMI significantly modified the influence of both MEP and triclosan on age of menarche. An increase in either biomarker was only associated with earlier menarche among girls that were either overweight or obese. Among U.S. children, neither association was significantly modified by BMI; however, the association with MEP was in a similar direction among all girls [25, 27]. It is possible that these differences in effect modification by BMI are due to race/ethnicity disparities in adolescent body composition [55]. Compared to white U.S. girls, Asian and Hispanic adolescents have greater trunk fat placement independent of total body fat [55]. Furthermore, Hispanic girls tend to have higher percent body fat than white girls, adjusting for body size [56]. It is possible the action of MEP and triclosan may be modified by elements of metabolic profile shaped by fat distribution, such as modulations in insulin resistance or leptin production [18, 57]. Alternatively, the observed statistical interaction with BMI may reflect an influence of MEP and triclosan on both adiposity and menarcheal age. Prenatal exposure to MEP has been associated with decreased childhood BMI Z-score [23], but this association has been inconsistent across populations [22]. In one cross-sectional study, triclosan exposure was similarly associated with a decrease in BMI and waist circumference among children [58], but this correlation was not observed in a second cross-sectional study [38]. Given the impact of childhood adiposity on pubertal timing [59–63], the potential influence of EDCs on adiposity raises important consideration of the direction of causation. Specifically, EDC exposure could have a direct effect on menarcheal age, or influence adiposity to impact pubertal timing, or influence pubertal timing to impact adiposity. These theories are not mutually exclusive, and should be addressed by follow-up studies.

One limitation to this study is that we cannot account for the influence of earlier EDC exposure on pubertal timing that may have partially confounded the observed associations due to consistent home environment. For example, there is indication that exposure to certain EDCs *in utero*, such as DEHP, may influence both childhood adiposity and pubertal timing [21–23, 28]. A second limitation is that while this study did measure urinary biomarkers at two developmental time points, concentrations in a single spot urine may not be representative of average exposure. Nonetheless, prior longitudinal assessments of EDC concentrations suggest that spot measurements can estimate relative EDC exposures over the course of several months to a year due to recurrent exposures [64–67]. A third important consideration is that these girls were primarily from low socioeconomic status families, which may have an exposure profile that is not representative of all children and adolescents. However, given the faster decline in menarcheal age among impoverished girls [6, 8, 9, 14, 15], the determinants of pubertal timing within this subset of the population is of particular public health concern. Finally, this study did not account for potential synergistic or antagonistic impacts of EDC co-exposures on pubertal timing. In addition to the studied biomarkers, exposure to other types of unmeasured EDCs, such as persistent organic pollutants, may have modified our observed associations with pubertal development. These interactions may contribute to the lack of reproducibility in the associations between EDC biomarkers and the onset of menarche across populations. Given the concurrence of EDCs in consumer products and the home environment [68], it is difficult to disentangle the independent influence of any single chemical on menarcheal age. However, we hope these findings spur additional research into modifiable sources of EDC exposure during childhood and adolescence.

## Conclusions

Exposure to certain phenols and phthalates during specific stages of pubertal development was associated with menarcheal age in Chilean girls. This study highlights the necessity of capturing critical windows of exposure during periods of rapid physiological change. Additionally, the unique associations observed in this cohort emphasize the need to integrate investigations from across the world to identify population-specific sensitivities, which can guide global health recommendations. As data collection in this longitudinal cohort continues, we plan to explore the impact of EDC exposure on additional facets of pubertal development, such as the onset of regular cycling. These shifts in pubertal timing may have both immediate psychological implications for these girls, and may indicate changes in the predisposition to adverse health in adulthood. Girls with early puberty have been shown to suffer from higher rates of adolescent depression, anxiety, and delinquent behavior [69–72]. Early onset of menarche has also been associated with increased risk of type-2 diabetes, cardiovascular disease, and cancer risk. Future studies will be necessary to directly link the variation in pubertal timing associated with early life EDC exposure to adult health, as well as the potential direct effect of childhood/adolescent EDC exposure on adult health independent of age of menarche.

## Additional file

Additional file 1: **Table S1a.** Phthalate metabolite urinary concentrations corrected for specific gravity in a Chilean girls cohort (*n* = 200). **Table S1b.** Phenol biomarker urinary concentrations corrected for specific gravity in a Chilean girls cohort (*n* = 200). **Table S2.** Geometric mean (95% CI) creatine-adjusted urinary EDC biomarker concentrations and age at measurement in the 2011–2012 U.S. National Health and Nutrition Examination Survey among females 5 to 14 years. **Table S3.** Spearman correlation between EDC SG-adjusted biomarker urinary concentrations (ng/ml) at B1 and B4 in Chilean cohort (*n* = 200). **Table S4.** Sensitivity analysis: menarche hazard ratio (95% CI) associated with log(ng/ml) increase in each EDC biomarker across puberty adjusting for mother's age of menarche. **Table S5.** Sensitivity analysis: menarche hazard ratio (95% CI) associated with log(ng/ml) increase in biomarker stratified by Tanner stage adjusting for mother's age of menarche. (DOCX 27 kb)

## Abbreviations

24-DCP: 2,4-dichlorophenol
25-DCP: 2,5-dichlorophenol
AFT: Accelerated failure time
B1: Tanner 1
B4: Tanner 4
BP-3: Benzophenone-3
BPA: Bisphenol A
B-PB: Butyl paraben
BPF: Bisphenol F
BPS: Bisphenol S
DEHP: of di (2-ethylhexyl) Phthalate
EDC: Endocrine disrupting chemical
E-PB: Ethyl paraben
MBP: Mono-n-butyl phthalate
MBzP: Monobenzyl phthalate
MCNP: Mono carboxyisononyl phthalate
MCOP: Mono carboxyisooctyl phthalate
MCPP: Mono-3-carboxypropyl phthalate
MECPP: Mono(2-ethyl-5-carboxypentyl) phthalate
MEHHP: Mono(2-ethyl-5-hydroxyhexyl) phthalate
MEHP: Mono(2-ethylhexyl) phthalate
MEOHP: Mono(2-ethyl-5-oxohexyl) phthalate
MEP: Monoethyl phthalate
MHBP: Mono-hydroxybutyl phthalate
MHiBP: Mono-hydroxyisobutyl phthalate
MiBP: Mono-isobutyl phthalate
MMP: Monomethyl phthalate
MNP: Mono-isononyl phthalate
M-PB: Methyl paraben
P-PB: Propyl paraben
TCS: Triclosan
