# Supplementary material for: Childhood and adolescent phenol and phthalate exposure and the age of menarche in Latina girls
Source: Environ Health. 2018 Apr 3;17:32. doi: 10.1186/s12940-018-0376-z (PMC5883544; doi:10.1186/s12940-018-0376-z)
Supplement: Supplementary file 1 — Table S1a. Phthalate metabolite urinary concentrations corrected for specific gravity in a Chilean girls cohort (n = 200). Table S1b. Phenol biomarker urinary concentrations corrected for specific gravity in a Chilean girls cohort (n = 200). Table S2. Geometric mean (95% CI) creatine-adjusted urinary EDC biomarker concentrations and age at measurement in the 2011–2012 U.S. National Health and Nutrition Examination Survey among females 5 to 14 years. Table S3. Spearman correlation between EDC SG-adjusted biomarker urinary concentrations (ng/ml) at B1 and B4 in Chilean cohort (n = 200). Table S4. Sensitivity analysis: menarche hazard ratio (95% CI) associated with log(ng/ml) increase in each EDC biomarker across puberty adjusting for mother's age of menarche. Table S5. Sensitivity analysis: menarche hazard ratio (95% CI) associated with log(ng/ml) increase in biomarker stratified by Tanner stage adjusting for mother's age of menarche. (DOCX 27 kb) [file 12940_2018_376_MOESM1_ESM.docx]

**SUPPLEMENTARY TABLES**

| **Supplementary Table 1a.** Phthalate metabolite urinary concentrations corrected for specific gravity in a Chilean girls cohort (n=200) | | | | | | |
| --- | --- | --- | --- | --- | --- | --- |
|  |  |  | **Tanner B1** | | **Tanner B4** | |
| **Biomarker** | Acronym | LOD (ng/ml) | Above LOD (%) | Geometric Mean (ng/ml; 95% CI) | Above LOD (%) | Geometric Mean (ng/ml; 95% CI) |
| Mono-n-butyl phthalate | MBP | 0.4 | 99.5 | 23.41  (20.62, 26.58) | 97 | 23.47  (20.58, 26.76) |
| Monobenzyl phthalate | MBzP | 0.3 | 97 | 3.13  (2.72, 3.60) | 92 | 2.26  (1.99, 2.58) |
| Mono carboxyisononyl phthalate | MCNP | 0.2 | 97.5 | 1.21  (1.07, 1.36) | 91 | 0.96  (0.86, 1.06) |
| Mono carboxyisooctyl phthalate | MCOP | 0.2 | 100 | 11.35  (9.95, 12.94) | 100 | 9.54  (8.55, 10.64) |
| Mono-3-carboxypropyl phthalate | MCPP | 0.2 | 98 | 1.48  (1.30, 1.68) | 91 | 1.63  (1.43, 1.85) |
| Mono(2-ethyl-5-carboxypentyl) phthalate | MECPP | 0.2 | 100 | 50.60  (45.91, 55.78) | 100 | 36.00  (32.39, 40.01) |
| Mono(2-ethyl-5-hydroxyhexyl) phthalate | MEHHP | 0.2 | 100 | 24.71  (22.22, 27.48) | 100 | 17.33  (15.43, 19.47) |
| Mono(2-ethylhexyl) phthalate | MEHP | 0.5 | 89.5 | 2.38  (2.13, 2.65) | 86 | 2.23  (1.98, 2.52) |
| Mono(2-ethyl-5-oxohexyl) phthalate | MEOHP | 0.2 | 100 | 15.05  (13.56, 16.70) | 99.5 | 11.21  (10.00, 12.56) |
| Monoethyl phthalate | MEP | 0.6 | 100 | 103.40  (88.63, 120.63) | 100 | 76.39  (64.97, 89.82) |
| Mono-hydroxybutyl phthalate | MHBP | 0.4 | 95 | 2.42  (2.15, 2.72) | 88.5 | 2.25  (2.02, 2.50) |
| Mono-hydroxyisobutyl phthalate | MHiBP | 0.4 | 100 | 9.70  (8.70, 10.82) | 98.5 | 9.94  (8.98, 11.01) |
| Mono-isobutyl phthalate | MiBP | 0.2 | 100 | 22.65  (20.32, 25.26) | 100 | 25.83  (23.22, 28.72) |
| Monomethyl phthalate | MMP | 0.5 | 84.5 | 1.74  (1.54, 1.97) | 81.5 | 2.06  (1.84, 2.30) |
| Mono-isononyl phthalate | MNP | 0.5 | 22.5 | 0.52  (0.47, 0.58) | 27 | 0.57  (0.52, 0.63) |

| **Supplementary Table 1b.** Phenol biomarker urinary concentrations corrected for specific gravity in a Chilean girls cohort (n=200) | | | | | | |
| --- | --- | --- | --- | --- | --- | --- |
|  |  |  | **Tanner B1** | | **Tanner B4** | |
| **Biomarker** | Acronym | LOD (ng/ml) | Above LOD (%) | Geometric Mean (ng/ml; 95% CI) | Above LOD (%) | Geometric Mean (ng/ml; 95% CI) |
| 2,4-dichlorophenol | 24-DCP | 0.1 | 98 | 0.62  (0.55, 0.70) | 96.5 | 0.62  (0.55, 0.70) |
| 2,5-dichlorophenol | 25-DCP | 0.1 | 100 | 3.20  (2.69, 3.79) | 98 | 2.36  (1.99, 2.79) |
| Butyl Paraben | B-PB | 0.1 | 55.5 | 0.35  (0.27, 0.44) | 51 | 0.28  (0.22, 0.35) |
| benzophenone-3 | BP-3 | 0.4 | 99 | 16.33  (13.39, 19.93) | 97.5 | 32.32  (25.37, 41.17) |
| bisphenol A | BPA | 0.2 | 95.5 | 0.99  (0.90, 1.09) | 93 | 1.12  (1.02, 1.24) |
| bisphenol F | BPF | 0.2 | 36.5 | 0.24  (0.22, 0.27) | 38.5 | 0.27  (0.24, 0.31) |
| bisphenol S | BPS | 0.1 | 66 | 0.20  (0.17, 0.23) | 64 | 0.22  (0.19, 0.26) |
| Ethyl Paraben | E-PB | 1 | 38 | 1.92  (1.59, 2.31) | 32 | 1.70  (1.42, 2.04) |
| Methyl Paraben | M-PB | 1 | 97.5 | 36.03  (28.24, 45.98) | 95 | 19.50  (15.47, 24.58) |
| Propyl Paraben | P-PB | 0.1 | 99 | 3.35  (2.55, 4.40) | 95 | 2.08  (1.62, 2.67) |
| triclosan | TCS | 1.7 | 86.5 | 7.37  (6.18, 8.78) | 88 | 11.00  (9.06, 13.36) |
|  | | | | | | |

| **Supplemental Table 2**. Geometric mean (95% CI) creatine-adjusted urinary EDC biomarker concentrations and age at measurement in the 2011-2012 U.S. National Health and Nutrition Examination Survey among females 5 to 14 years | | | |
| --- | --- | --- | --- |
|  |  | Geometric Mean (μg/g Creatine; 95% CI) | |
| EDC | Above LOD (%) | Ages 6-9 | Ages 10-13 |
| B-PB | 29.8 | 0.35 (0.29, 0.43) | 0.29 (0.23, 0.37) |
| BP-3 | 99.18 | 23.84 (17.76, 32.01) | 19.59 (14.70, 26.09) |
| BPA | 91.84 | 2.08 (1.83, 2.37) | 1.82 (1.58, 2.08) |
| E-PB | 35.51 | 2.30 (1.84, 2.87) | 1.47 (1.17, 1.85) |
| M-PB | 97.96 | 50.15 (37.53, 67.01) | 53.92 (40.12, 72.46) |
| MBP | 95.92 | 21.16 (17.57, 25.48) | 15.84 (13.52, 18.55) |
| MBzP | 100 | 10.40 (8.68, 12.47) | 7.65 (6.47, 9.05) |
| MCNP | 100 | 4.06 (3.55, 4.63) | 2.90 (2.57, 3.28) |
| MCOP | 100 | 28.10 (23.94, 32.97) | 22.74 (19.13, 27.04) |
| MCPP | 99.59 | 4.80 (4.14, 5.56) | 3.58 (2.95, 4.36) |
| MECPP | 99.59 | 33.44 (29.61, 37.78) | 19.41 (16.75, 22.49) |
| MEHHP | 100 | 18.17 (15.75, 20.96) | 11.46 (9.92, 13.24) |
| MEHP | 80.41 | 2.29 (2.00, 2.62) | 1.74 (1.48, 2.05) |
| MEOHP | 100 | 12.21 (10.68, 13.96) | 7.64 (6.67, 8.76) |
| MEP | 100 | 35.48 (28.92, 43.53) | 29.16 (23.59, 36.04) |
| MHiNCH | 50.61 | 0.94 (0.82, 1.08) | 0.47 (0.41, 0.54) |
| MiBP | 99.59 | 14.68 (12.60, 17.10) | 11.09 (9.71, 12.67) |
| MMP | 77.55 | 3.80 (2.97, 4.85) | 2.19 (1.76, 2.73) |
| MNP | 61.22 | 2.06 (1.71, 2.47) | 1.98 (1.61, 2.43) |
| P-PB | 97.96 | 6.91 (4.96, 9.62) | 6.90 (4.94, 9.64) |
| TCS | 71.84 | 11.15 (8.58, 14.49) | 10.77 (7.85, 14.76) |

| **Supplementary Table 3.** Spearman correlation between EDC SG-adjusted biomarker urinary concentrations (ng/ml) at B1 and B4 in Chilean cohort (n=200) | | |
| --- | --- | --- |
| EDC | Rho | p-value |
| 2,4-DCP | 0.217 | 2.01E-03 |
| 25-DCP | 0.152 | 3.19E-02 |
| B-PB | 0.185 | 8.80E-03 |
| BP-3 | 0.103 | 1.45E-01 |
| BPA | 0.184 | 9.20E-03 |
| BPF | -0.012 | 8.71E-01 |
| BPS | 0.045 | 5.27E-01 |
| E-PB | 0.079 | 2.67E-01 |
| M-PB | 0.093 | 1.88E-01 |
| MBP | 0.142 | 4.44E-02 |
| MBzP | 0.330 | 1.84E-06 |
| MCNP | 0.079 | 2.63E-01 |
| MCOP | 0.133 | 6.08E-02 |
| MCPP | 0.111 | 1.16E-01 |
| MECPP | 0.221 | 1.68E-03 |
| MEHHP | 0.166 | 1.86E-02 |
| MEHP | 0.302 | 1.40E-05 |
| MEOHP | 0.193 | 6.16E-03 |
| MEP | 0.325 | 2.75E-06 |
| MHBP | 0.111 | 1.18E-01 |
| MHiBP | 0.330 | 1.86E-06 |
| MiBP | 0.279 | 6.22E-05 |
| MMP | -0.002 | 9.78E-01 |
| MNP | 0.194 | 5.94E-03 |
| P-PB | 0.117 | 9.78E-02 |
| TCS | 0.146 | 3.96E-02 |

| **Supplemental Table 4**. Sensitivity analysis: menarche hazard ratio (95% CI) associated with log(ng/ml) increase in each EDC biomarker across puberty adjusting for mother’s age of menarche | | | |
| --- | --- | --- | --- |
| Biomarker | Unadjusted Model | Adjusted Model^a^ | Interaction with Tanner^b^ |
| 2,4-Dichlorophenol | 0.98 (0.87, 1.10) | 0.99 (0.88, 1.12) | 0.653 |
| 2,5-Dichlorophenol | 1.05 (0.97, 1.14) | 1.03 (0.94, 1.13) | 0.020* |
| Benzophenone-3 | 1.03 (0.97, 1.09) | 1.05 (1.00, 1.12) | 0.034 |
| BPA | 0.90 (0.78, 1.05) | 0.93 (0.80, 1.08) | 0.790 |
| MBP | 0.95 (0.84, 1.07) | 0.95 (0.84, 1.07) | 0.575 |
| MBzP | 0.98 (0.87, 1.09) | 0.95 (0.84, 1.06) | 0.802 |
| MCNP | 0.95 (0.82, 1.10) | 0.90 (0.79, 1.03) | 0.361 |
| MCOP | 0.95 (0.81, 1.10) | 0.90 (0.78, 1.05) | 0.808 |
| MCPP | 0.91 (0.80, 1.04) | 0.91 (0.80, 1.02) | 0.355 |
| ΣDEHP^c^ | 1.04 (0.88, 1.22) | 1.02 (0.87, 1.19) | 0.002** |
| MECPP | 1.05 (0.89, 1.24) | 0.98 (0.84, 1.14) | 0.008** |
| MEHHP | 1.03 (0.89, 1.18) | 0.96 (0.84, 1.10) | 0.004** |
| MEHP | 1.01 (0.85, 1.20) | 0.95 (0.81, 1.11) | 0.005** |
| MEOHP | 1.02 (0.88, 1.19) | 0.95 (0.83, 1.10) | 0.007** |
| MEP | 1.10 (0.99, 1.23) | 1.11 (0.99, 1.23) | 0.085 |
| Methyl Paraben | 1.00 (0.94, 1.07) | 0.99 (0.93, 1.05) | 0.820 |
| MHBP | 1.02 (0.89, 1.16) | 1.00 (0.88, 1.14) | 0.514 |
| MHiBP | 1.04 (0.88, 1.23) | 1.04 (0.89, 1.21) | 0.402 |
| MiBP | 1.02 (0.87, 1.19) | 1.01 (0.87, 1.18) | 0.951 |
| MMP | 1.05 (0.93, 1.19) | 1.10 (0.98, 1.23) | 0.092 |
| Propyl Paraben | 0.99 (0.92, 1.05) | 0.98 (0.93, 1.04) | 0.976 |
| Triclosan | 0.98 (0.90, 1.07) | 0.99 (0.91, 1.07) | 0.468 |
| ^a^Accelerated failure time model adjusting for BMI Z-score, maternal education, and maternal age of menarche among the subset with maternal age of menarche data (N=181)  ^b^p-value for interaction between continuous biomarker concentration and tanner stage in adjusted models  ^c^units in log(nmol/l) reflecting the log transformed summation of DEHP metabolite concentrations  *p<0.05, **p<0.01, ***p<0.001 | | | |

| **Supplemental Table 5**. Sensitivity analysis: menarche hazard ratio (95% CI) associated with log(ng/ml) increase in biomarker stratified by Tanner stage adjusting for mother’s age of menarche^a^ | | | |
| --- | --- | --- | --- |
|  | | Tanner Stage | |
| Biomarker | B1 | | B4 |
| 2,5-Dichlorophenol | 1.10 (0.99, 1.23) | | 0.96 (0.86, 1.06) |
| Benzophenone-3 | 1.16** (1.05, 1.28) | | 1.01 (0.93, 1.09) |
| ΣDEHP^b^ | 0.77* (0.60, 0.98) | | 1.24 (0.97, 1.57) |
| MECPP | 0.78* (0.62, 0.98) | | 1.14 (0.90, 1.45) |
| MEHHP | 0.76** (0.62, 0.93) | | 1.13 (0.92, 1.40) |
| MEHP | 0.79* (0.65, 0.97) | | 1.08 (0.89, 1.31) |
| MEOHP | 0.76** (0.62, 0.94) | | 1.11 (0.89, 1.37) |
| MMP | 1.00 (0.84, 1.18) | | 1.25* (1.05, 1.48) |
| ^a^Accelerated failure time model adjusting for BMI Z-score, maternal age of menarche, and maternal education; including an interaction between Tanner stage at biomarker measurement and concentration; restricted to subset of associations for which the interaction with the timing of biomarker measurement was significant (p<0.05); among the subset of girls with maternal age of menarche data (N=181)  ^b^units in log(nmol/l) reflecting the log transformed summation of DEHP metabolite concentrations  *p<0.05, **p<0.01, ***p<0.001 | | | |
